# A Distinct Group II Alphabaculovirus Isolated from a Peridroma Species

**DOI:** 10.1128/genomeA.00185-15

**Published:** 2015-04-02

**Authors:** George F. Rohrmann, Martin A. Erlandson, David A. Theilmann

**Affiliations:** a Department of Microbiology, Oregon State University, Corvallis, Oregon, USA; b Saskatoon Research Centre, Agriculture and Agri-Food Canada, Saskatoon, Saskatchewan, Canada; c Pacific Agri-Food Research Centre, Agriculture and Agri-Food Canada, Summerland, British Columbia, Canada

## Abstract

The genome sequence of an alphabaculovirus isolated from a Peridroma species indicated that it is a novel member of a group II lineage most closely related to alphabaculoviruses from Spodoptera exigua and Agrotis segetum. It contains a genome of 151,110 nucleotides (nt), with a G+C content of 53.3%.

## GENOME ANNOUNCEMENT

A baculovirus isolated from a cutworm larvae (Peridroma species) (Lepidoptera: Noctuidae), which we have called Peridroma sp. nucleopolyhedrovirus (PespNPV), is part of an archival collection of infected insects (1). A partial *lef8* gene sequence indicates that it is a member of a group II lineage of alphabaculoviruses and is significantly different from other members of that lineage (1). In order to further characterize this virus, the genome was sequenced and determined to be 151,110 bp, with a G+C content of 53.3%, containing 138 predicted open reading frames (ORFs) of ≥25 amino acids. The orthologs of all 37 baculovirus core genes were identified.

The relatedness of baculovirus genomes is normally determined by comparing the PIF2 and LEF8 amino acid sequences. The sequences of both these genes from PespNPV showed the closest relatedness to sequences from the group II lineage, which includes Agrotis segetum NPV and Spodoptera exigua NPV, with amino acid sequence identities of 80 and 77% (for PIF2) and 82 and 77% (for LEF8), respectively. Overall, the homologies to other known baculoviruses suggests that PespNPV belongs to a distinct alphabaculovirus species.

The PespNPV genome has 6 ORFs not found in other baculoviruses. These include pesp-005, -021, -034, -076, -113, and -138. Five of these ORFs also lacked similar ORFs in GenBank; however, ORF138 is related to uncharacterized ORFs from ascoviruses (BLAST expect value, 3e-12) and iridoviruses. Two ORFs (pesp-095 and -097) have 40% amino acid sequence identity, suggesting that they resulted from a gene duplication. They are also related to an uncharacterized ORF from Danaus plexippus, the monarch butterfly (BLAST expect value, 9E-17).

A feature of many baculovirus genomes is homologous repeat sequences (*hrs*) that often have a palindromic structure (2). The PespNPV sequence was observed to have one major *hr* located at nucleotides (nt) 16586 to 17726. It contains 8 homologous repeats that are 32 nt long, with the consensus sequence GAATTCAYCGACRATTRTCRRYGAACGRCANN, and 24 of the nucleotides are invariant among the 8 repeats. A 22-nt subsequence of the *hr*, TTCAYCGACRATTRTCRRYGAA, forms a palindrome with up to 5 mismatches. Although the *hrs* start with an EcoR1 site (GAATTC), only the TTC is included in the palindromes. There are single repeats of this *hr* sequence at nt 51090, 10890, and 134736.

### Nucleotide sequence accession number.

The genome sequence of PespNPV has been deposited in GenBank under the accession no. KM009991.

## References

[1] Rohrmann GF 2013 Characterization of baculoviruses from the Martignoni collection. J Invertebr Pathol 114:61–64. doi:10.1016/j.jip.2013.04.005. 23628143

[2] Rohrmann GF 2013 Baculovirus molecular biology. National Center for Biotechnology Information, Bethesda, MD. 24479205

